# Propionic acid mediates the renoprotective effects of fecal microbiota transplantation against ischemia–reperfusion injury via upregulating GPR43

**DOI:** 10.3389/fcimb.2025.1616164

**Published:** 2025-09-25

**Authors:** Jingxuan Yu, Zhenyu Liu, Yan Wang, Yu Zhou, Wei Liu, Tao Wang, Qiubo Xie, Hongzhe Tian, Yalong Xu, Min Wang, Fuhan Zhao, Lin Wang, Guan Zhang, Dongliang Chen, Lei Gao, Tiejun Pan

**Affiliations:** ^1^ Department of Urology, General Hospital of Central Theater Command of Chinese People’s Liberation Army, Wuhan, Hubei, China; ^2^ School of Medicine, Wuhan University of Science and Technology, Wuhan, Hubei, China; ^3^ China Peptide and Life Science Research Institute, Wuhan, Hubei, China; ^4^ China Future Food Innovation Institute, Wuhan, Hubei, China

**Keywords:** fecal microbiota transplantation, acute kidney injury, ischemia-reperfusion injury, short-chain fatty acids, Lachnospiraceae

## Abstract

**Introduction:**

Kidney ischemia–reperfusion injury (IRI) is a major cause of acute kidney injury (AKI), characterized by aggravated inflammation and apoptosis following reperfusion. This study aimed to investigate the protective effects and mechanisms of fecal microbiota transplantation (FMT) in a rat model of kidney IRI.

**Methods:**

Sprague–Dawley rats(SDRs) subjected to 45 minutes of bilateral renal ischemia followed by reperfusion were prophylactically treated with FMT derived from guinea pigs or supplemented with propionic acid. Renal function, histopathology, inflammatory markers, apoptosis, proliferation, and gut microbiota composition were systematically evaluated.

**Results:**

The results demonstrated that FMT attenuated kidney IRI by remodeling the gut microbiota to enhance propionic acid production, which subsequently modulated inflammation and apoptosis via GPR43 signaling.

**Conclusions:**

These findings provide novel insights into microbiota-targeted therapeutic strategies for kidney IRI and highlight propionic acid as a potential therapeutic agent.

## 1 Introduction

Acute kidney injury (AKI) is a global healthcare burden, affecting 20%–25% of hospitalized patients and contributing to high mortality rates (1.7- to 3.3-fold increase) and progression to chronic kidney disease (CKD) (Allinson et al., 2023; Huang et al., 2023; Song and Gong, 2023; Li et al., 2024b). Kidney ischemia–reperfusion injury (IRI), a leading cause of AKI, triggers inflammatory response and tubular apoptosis. However, targeted therapies remain unavailable (Daemen et al., 2002; Huang et al., 2022). Emerging evidence implicates the gut–kidney axis in kidney pathology, where dysbiosis—marked by a reduced microbial diversity and the loss of beneficial taxa—exacerbates systemic inflammation and kidney dysfunction (Vaziri, 2012; Ramezani and Raj, 2014; Chen et al., 2019). Specifically, AKI-associated gut dysbiosis disrupts the intestinal barrier integrity, promoting bacterial translocation and endotoxemia that amplify kidney injury (Ramezani et al., 2016; Yang et al., 2020).

Fecal microbiota transplantation (FMT), an intervention proven effective against *Clostridioides difficile* infection and metabolic disorders (Wang et al., 2019), has recently demonstrated therapeutic potential in kidney diseases (Kim et al., 2023; Miao et al., 2024a, 2024; Shen and Su, 2024; Xiao et al., 2025). In the majority of cases, this therapy is used to treat gastrointestinal disorders caused by the activity of pathogenic or conditionally pathogenic microorganisms. Beyond gastrointestinal restoration, the therapeutic reach of FMT now extends to multisystem pathologies, including diabetes remission strategies, antitumor immune potentiation, and α-synucleinopathy mitigation in Parkinson’s disease, as evidenced by transformative cohort studies (Sampson et al., 2016; Riquelme et al., 2019; Wu et al., 2022). Building on prior methodology (Wang et al., 2023), we established that FMT from guinea pigs to Sprague–Dawley rats (SDRs) ameliorates calcium oxalate nephrolithiasis by reconstructing the gut microbiota functional networks critical for oxalate metabolism. Inspired by this study, we aimed to investigate whether FMT could exert a protective effect on kidney IRI and to further elucidate its underlying mechanism. In addition, we used enteric-coated capsules instead of the traditional FMT method to reduce the impact of gastric acid on the microbial community.

Lachnospiraceae, in the gut of majority of healthy individuals, may be a beneficial bacterium. It participates in the metabolism of various carbohydrates and exhibits particularly strong capabilities in metabolizing pectin (a complex dietary fiber and prebiotic), particularly the pectin derived from fruits and vegetables. Its fermentation process leads to the production of short-chain fatty acids (SCFAs).

SCFAs—microbial metabolites such as acetate, propionate, and butyrate—are key mediators of the gut–kidney crosstalk (Huang et al., 2017), and they have a series of effects such as anti-inflammatory and anti-apoptosis, among others. SCFAs not only can act locally in organs (Ratajczak et al., 2019; Ney et al., 2023) but also can reach the bloodstream (Cummings et al., 1987). SCFAs attenuate kidney injury by suppressing the NF-κB-driven inflammation and oxidative stress (Andrade-Oliveira et al., 2015; Sun et al., 2022). They can also signal through G protein-coupled receptors (GPRs), such as GPR41 and GPR43, to regulate inflammatory responses and apoptosis (Kim et al., 2013; Behler-Janbeck et al., 2024). However, more in-depth research is needed to determine whether SCFAs can function through GPRs. Therefore, in this study, we hypothesize that prophylactic FMT enriches Lachnospiraceae to increase propionic acid, activating GPR43 to suppress the NF-κB-driven inflammation/apoptosis and mitigate renal IRI.

IRI represents a significant threat to kidney function, potentially leading to AKI or even complete kidney failure in patients undergoing kidney transplantation. Despite its severity, effective therapeutic interventions for IRI are currently lacking. Prophylactic FMT therapy has emerged as a promising approach that may mitigate the effects of IRI. Nevertheless, the specific mechanisms and pathways through which FMT exerts its beneficial effects in the context of IRI remain inadequately understood.

## 2 Materials and methods

### 2.1 Experimental model

The SDRs (males, 250–300 g) and guinea pigs (males, 300–350 g) were purchased from the Experimental Animal Experiment Center of Wuhan University. The protocols for the animal experiments were approved by the Animal Ethics Committee of Wuhan University (WP20240357 and WP20220064). All animals were housed in a laminar atmosphere without specific pathogen (specific pathogen-free, SPF). The animal experiments were conducted according to the guidelines of the National Institutes of Health in the United States and were fully authorized by the Experimental Animal Center of Wuhan University. All animals were kept at 27 ± 2°C with a 12-h light/dark cycle.

### 2.2 Rat model of kidney IRI

The rat IRI models were constructed as described below (Andrade-Oliveira et al., 2015; Zou et al., 2021). The SDRs were anesthetized with chloral hydrate, with a midline incision made, and two sides of the kidney hilum were clamped for 45 min. During this procedure, the animals were kept well hydrated with saline through a heating pad device and maintained at a constant temperature (∼37°C). Subsequently, the microsurgical clamp was removed, the abdomen was closed, and the animals were placed in individual cages and heated with indirect light until complete recovery from anesthesia. The animals were placed under adjustable conditions until execution, i.e., 24 h after kidney reperfusion. Blood samples were collected through an inferior vena cava puncture and fresh kidney tissue collected. Serum was separated from the blood samples by centrifugation at 4,500 × *g* for 10 min and kept at −80°C. The whole kidney tissue from each rat was divided into two parts and fixed in 4% paraformaldehyde or stored at −80°C.

### 2.3 FMT experiments

There were six SDRs per group of the control (Ctrl) group, the IRI group (where kidney IRI modeling was performed in SDRs), the normal saline (NS) group (with 0.9% saline), the FMT group (with fecal microbiota transplantation), the IRI+FMT group (where kidney IRI modeling was performed in SDRs after FMT was completed), and the IRI+NS group (after gavage with normal saline, kidney IRI modeling was performed in SDRs).

### 2.4 Propionic acid treatment experiment

There were six SDRs per group. For the propionic acid (Prop) group, 100 mM sodium propionate (Sigma-Aldrich, St. Louis, MO, USA) was dissolved and administered *ad libitum* in drinking water. Controls received pH- and sodium-matched water. The dietary and drinking solutions were replenished three times per week. The SDRs in the IRI+Prop group were randomized to receive propionate solutions for 3 weeks and then underwent kidney IRI modeling. The SDRs in the IRI+NS group were randomized to receive pH- and sodium-matched water for 3 weeks and then underwent kidney IRI modeling.

### 2.5 FMT and preparation of gavage capsule

The FMT capsules were produced as described below (Zain et al., 2022). Fresh stool (4 g/batch) from guinea pig underwent pathogen testing (e.g., *C. difficile*, parasites, and viruses). Fecal samples with reduced moisture content were collected using metabolic cages, followed by cryopreservation at −80°C for 24 h. The frozen specimens were subsequently pulverized into a homogeneous powder, which was then encapsulated in enteric-coated capsules (0.025 ml capacity, 2.71 mm maximum outer diameter, 23.0 mm maximum locking length) (**Table 1**) designed for gastric acid resistance and intestinal release. Each capsule contained 30 mg of powdered material. SDRs received oral administration twice daily via a sterile intubation device, with two capsules per dose administered consecutively for 7 days.

**Table 1:** Key resource table.

Reagent or resource	Source	Identifier
Antibodies		
PCNA cell proliferation detection kit (IHC)	Beijing Biolab Technology, Beijing, China	Cat. no. KFS333, RRID: AB_3674660
Beta-actin antibody	GeneTex, San Antonio, TX, USA	Cat. no. GTX21801, RRID: AB_1240407
GPR41 antibody	Affinity Biosciences, USA	Cat. no. AF9075, RRID: AB_2843266
FFAR2/GPR43 antibody	Novus, St. Louis, Missouri, USA	Cat. no. NBP3-12190, RRID: AB_3586322
IKB alpha antibody [E130]	Abcam, Shanghai, China	Cat. no. ab32518, RRID: AB_733068
Phospho-IKB alpha (Tyr42) polyclonal antibody	Thermo Fisher Scientific, Waltham, Massachusetts, USA	Cat. no. PA5-105545, RRID: AB_2816973
IL-1-alpha	RayBiotech, Guangzhou, China	Cat. no. DS-PB-01062, RRID: AB_854330
MCP-1 antibody	Abcam, Shanghai, China	Cat. no. ab7202, RRID: AB_305755
Anti-TNF-alpha/TNFA antibody	Boster Biological Technology, Pleasanton, USA	Cat. no. PB0082, RRID: AB_2811278
Anti-IL-6 antibody [1–6]	Huabio, Hangzhou, China	Cat. no. EM1701-45, RRID: AB_3068769
Chemicals, peptides, and recombinant proteins		
TRIzol	Invitrogen, California, USA	Cat. no. 15596018
Sodium propionate	Aladdin, Shanghai, China	Cat. no. S100121
Critical commercial assays		
NEXTFLEX^®^ Rapid DNA-Seq Kit 2.0	Bluescape	Cat. no. NOVA-5188-01
DNeasy PowerLyzer PowerSoil Kit	QIAGEN, Hilden, Germany	Cat. no. 12855-50
Rat blood urea nitrogen (BUN) ELISA kit	JSBOSSEN, Jiangsu, China	Cat. no. BS-E19309H2
Rat creatinine (Cr) ELISA kit	JSBOSSEN, Jiangsu, China	Cat. no. BS-E11216R2
Rat monocyte chemotactic protein 1 (MCP-1) ELISA kit	JSBOSSEN, Jiangsu, China	Cat. no. BS-E11050R2
Rat interleukin 6 (IL-6) ELISA kit	JSBOSSEN, Jiangsu, China	Cat. no. BS-E10980R2
Rat interleukin 1α (IL-1α) ELISA kit	JSBOSSEN, Jiangsu, China	Cat. no. BS-E10947R2
Rat tumor necrosis factor alpha (TNF-α) ELISA kit	JSBOSSEN, Jiangsu, China	Cat. no. BS-E12215R2
TUNEL *in situ* apoptosis kit (HRP-DAB method)	Elabscience, Wuhan, China	E-CK-A331
Experimental models: organisms/strains		
Guinea pigs	Experimental Animal Experiment Center of Wuhan University	N/A
Sprague–Dawley rats	Experimental Animal Experiment Center of Wuhan University	N/A
Oligonucleotides		
Primers (see *Section 2* for details)	This paper	N/A
Software and algorithms		
ImageJ	ImageJ software	Version1.51
GraphPad Prism	GraphPad software	Version
Photoshop	Adobe	N/A
QIIME2	https://qiime2.org/	version 2020.2
fastp	https://github.com/OpenGene/fastp	version 0.19.6
FLASH	http://www.cbcb.umd.edu/software/flash	version 1.2.11
mothur	http://www.mothur.org/wiki/Calculators	N/A
SourceTracker	https://github.com/danknights/sourcetracker	N/A
LEfSe analysis	http://huttenhower.sph.harvard.edu/LEfSe	N/A
MaAsLin2 software	https://github.com/biobakery/Maaslin2	N/A
Others		
Enteric-coated capsules	https://www.yuyanbio.com/geiyao/303.html	N/A

*LEfSe*, linear discriminant analysis effect size; *N/A*. not applicable.

### 2.6 Fecal manure collection and microbiome analysis

Feces were collected from SDRs housed in metabolic cages 14 days after fecal transplantation. The feces were frozen at −80°C until DNA extraction, and the fecal samples were extracted using the DNeasy PowerLyzer PowerSoil Kit (**Table 1**) to obtain genomic DNA. Subsequently, the extracted genomic DNA was detected with 1% agarose gel electrophoresis. The highly variable region (V4) of the bacterial 16S rRNA gene was amplified using primers 341F and 806R, and the PCR products were quantified with the QuantiFluor™-ST Blue Fluorescence Quantification System (Promega, Madison, WI, USA) based on the preliminary quantification results of the electrophoresis and then mixed according to the sequencing volume required for each sample. Library construction of the purified PCR products used the NEXTFLEX Rapid DNA-Seq Kit. Finally, the Illumina Nextseq2000 platform (Shanghai Meiji Bio-pharmaceutical Technology Co., Shanghai, China) was used for sequencing.

### 2.7 Amplicon sequence processing and analysis

After demultiplexing, the resulting sequences were quality filtered with fastp (0.19.6) and merged with FLASH (v1.2.11). Afterward, the high-quality sequences were denoised using the DADA2 (deblur) plug-in in the QIIME2 (version 2020.2) pipeline with the recommended parameters, which obtains single nucleotide resolution based on the error profiles within samples. DADA2 denoised sequences are commonly called amplicon sequence variants (ASVs). To minimize the effects of sequencing depth on the alpha and beta diversity measures, the number of sequences from each sample was rarefied to 20,000, which still yielded an average Good’s coverage of 97.90%. Taxonomic assignment of the ASVs was performed using the naive Bayes ++ consensus taxonomy classifier implemented in QIIME2 and the SILVA 16S rRNA database (v138). The metagenomic function was predicted with PICRUSt2 (Phylogenetic Investigation of Communities by Reconstruction of Unobserved States) based on the ASV representative sequences.

### 2.8 Enzyme-linked immunosorbent components

Blood was collected from rats, and the serum was separated and used to assess kidney function and inflammatory response using a double-antibody one-step sandwich enzyme-linked immunosorbent assay (ELISA) (**Table 1**) for the measurement of interleukin 1 alpha (IL-1α), monocyte chemoattractant protein 1 (MCP-1), tumor necrosis factor alpha (TNF-α), IL-6, blood urea nitrogen (BUN), and creatinine (Cr). To the coated wells of a pre-coated antibody, the specimen, the standard, and the horseradish peroxidase (HRP)-labeled detection antibody were added sequentially, incubated, and washed thoroughly. The color was developed with the TMB substrate, which was converted to blue by catalysis of peroxidase and to the final yellow color by acid. The shade of the color is positively correlated with the sample. Absorbance (optical density, OD) was measured at 450 nm using an enzyme marker, and the concentration of the sample was calculated.

### 2.9 Apoptosis assessment

Apoptosis was detected in the kidney using terminal deoxynucleotidyl transferase dUTP nick-end labeling (TUNEL) *In Situ* Apoptosis Kit (the HRP-DAB method) (**Table 1**) according to the manufacturer’s protocol. TUNEL-positive cells were counted through six random areas of each slide.

### 2.10 Histological evaluation

At the end of the experimental period, kidneys were removed from rats and were cut in half in the sagittal section. The kidney tissues were fixed in 10% formalin buffer and paraffin-embedded. Kidney paraffin sections (5 μm) were prepared and stained with hematoxylin and eosin (H&E) and were detected using light microscopy (×200). Injuries were graded on a five-point scale (Guo et al., 2023): 0 for normal kidney; 1 for minimal injury (<5% involvement of the cortex or the outer medulla); 2 for mild injury (5%–25% involvement of the cortex or the outer medulla); 3 for moderate injury (25%–75% involvement of the cortex or the outer medulla); and 4 for severe injury (approximately 75% involvement of the cortex or the outer medulla).

### 2.11 Immunohistochemistry

The kidney tissues were fixed in 4% paraformaldehyde, paraffin-embedded, and sectioned at 3 μm. The sections were baked (60°C, 60 min), dewaxed in xylene, and hydrated through an ethanol series. Antigen retrieval was performed in acidic buffer, followed by endogenous peroxidase blockade with 3% H_2_O_2_–methanol. After blocking nonspecific sites, the sections were incubated with anti-proliferating cell nuclear antigen (PCNA; 1:100) (**Table 1**) and an HRP-conjugated secondary antibody. DAB was used for signal development, counterstained with hematoxylin. Images were acquired under an Olympus BX51 microscope (×200).

### 2.12 RNA isolation and real-time PCR

Total RNA was extracted from the kidney tissue using TRIzol (**Table 1**). cDNA (2 μg total RNA) was synthesized using Moloney murine leukemia virus reverse transcriptase (Promega, Madison, WI, USA). TaqMan real-time fluorescence PCR was performed to quantify the mRNA levels of IL-6, IL-1α, TNF-α, MCP-1, GPR41, and GPR43 in the kidney. The primer sequences were as follows: forward, 5'-CCAGTATATACCACTTCACAAGTCGGA-3'; reverse, 5'-CAAGATGAGTTGGATGGTCTTGGTC-3', forward, 5'-CGTCAAGCAGGAGTTCATCA-3'; reverse, 5'-TTCTCCCTGAGCACTCACAA-3'; forward, 5'-GCCTCTTCTCATTCCTGCTT-3'; reverse, 5'-TGGGAACTTCTCATCCCTTTG-3'; forward, 5'-TAGCATCCACGTGCTGTCTC-3'; reverse, 5'-TGCTGCTGGTGATTCTCTTG-3'; forward, 5'-GTGGCCTTCTTTGAGTTCGGTG-3'; reverse, 5'-ATCCCAGCCTCCGTTATCCTG-3'; forward, 5'-TCTGCTCCTCTTCCTGCCATTCC-3'; reverse, 5'-CGTTCTATGCTCACCGTCATCAGG-3'; forward, 5'-TGCACCATCGTCATCATCGTTCAG-3'; reverse, 5'-ACCAGGCACAGCTCCAGTCG-3'.

### 2.13 Western blot

Kidney tissue extracts were prepared in RIPA lysis buffer supplemented with protease/phosphatase inhibitors, and the protein concentrations were determined using the BCA assay (Thermo Scientific, Waltham, MA, USA). For immunoblotting, 50 μg of the total protein per sample was separated by SDS-PAGE, transferred to membranes, and probed with the following primary antibodies (**Table 1**): IκBα (1:1,000), p-IκBα (1:1,000), GPR43 (1:200), GPR41 (1:200), IL-1α (1:200), MCP-1 (1:1,000), TNF-α (1:1,000), and IL-6 (1:500). β-Actin (1:1,000) served as the loading control. Protein bands were visualized using the Bio-Rad electrophoresis system and were quantified with Image Lab 3.0 software, with densities normalized to β-actin.

### 2.14 SCFA measurements

¹H-NMR spectroscopy was used to analyze the metabolic profiles in the fecal and serum samples. Fecal pellets were processed by bead-beating (5 m/s, 40 s) in ice-cold D_2_O and then centrifuged (16,000 × *g*, 5 min, 4°C). The supernatants from the fecal and serum samples were filtered (3 kDa cutoff) (Amicon Ultra), buffered with 200 mM Na_3_PO_4_/D_2_O and 5.0 mM DSS/D_2_O (pH 7.0), and transferred into 3-mm NMR tubes. Spectra were recorded at 298 K on a 600-MHz Bruker AVANCE III HD spectrometer with a cryoprobe. The Chenomx NMR Suite v8.4 was utilized for spectral processing (phase correction and baseline adjustment) and metabolite quantification, with DSS as the internal reference.

### 2.15 Quantification and statistical analysis

GraphPad Prism 9.0 (GraphPad Software, San Diego, CA, USA) was used for statistical analysis of the data. Data are expressed as the mean ± standard deviation. Normal distribution of the data was verified using the Shapiro–Wilk normality test. Depending on the normal distribution of the data, an unpaired *t*-test or the Mann–Whitney *U* test was used to compare two groups. ANOVA with Tukey’s *post-hoc* test was used for multiple comparisons. All comparisons were two-tailed, and a *p* < 0.05 was considered statistically significant. The software mothur (Schloss et al., 2009) (http://www.mothur.org/wiki/Calculators) was used to calculate the alpha diversity, Shannon’s index, and others. Principal coordinates analysis (PCoA) based on the Bray–Curtis distance algorithm was used to assess the similarity of the microbial community structure between samples. The SourceTracker software (https://github.com/danknights/sourcetracker) was used to show the species composition of the first N and the percentage of the community bar plot with different species (Knights et al., 2011). Linear discriminant analysis effect size (LEfSe) (Segata et al., 2011) (http://huttenhower.sph.harvard.edu/LEfSe) was used to identify the bacterial taxa with significant differences in abundance from the phylum to the genus level between groups. MaAsLin2 software (Mallick et al., 2021) (https://huthttps://huttenhower.sph.harvard.edu/maaslin/) was used to analyze the correlation heatmap plots representing the correlation between the species and the clinical factors, visualizing the magnitude of the correlation between multiple clinical factors and the different species and whether the difference in the correlation was significant.

## 3 Results

### 3.1 FMT attenuated kidney dysfunction and histopathological damage in IRI rats

**Figure 1:**
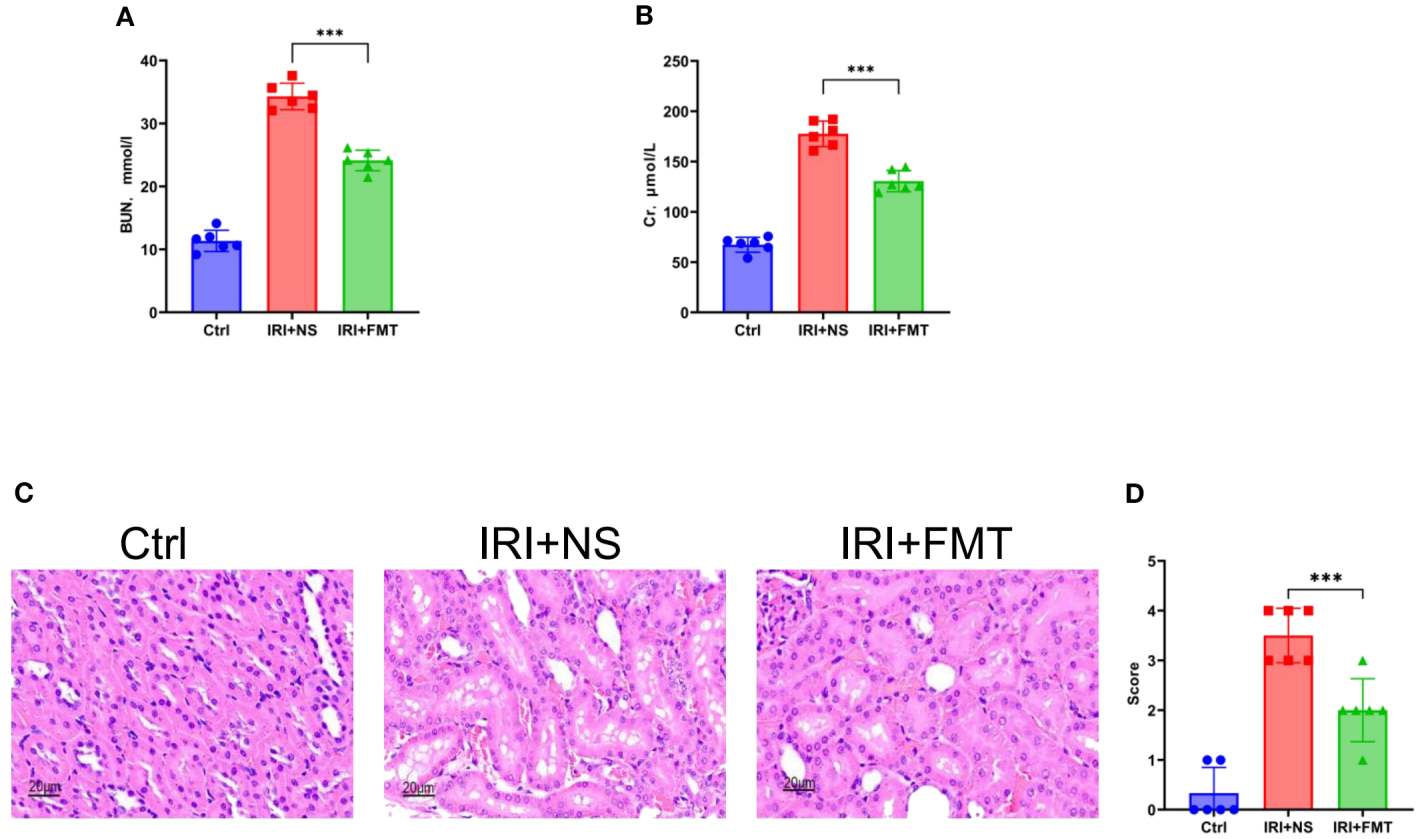
Fecal microbiota transplantation (FMT) improved kidney function. **(A)** Serum urea nitrogen in rats. **(B)** Serum creatinine in rats. **(C)** Hematoxylin–eosin (H&E) staining of the rats’ kidney sections. *Bar*, 20 μm. **(D)** Pathological scoring of the H&E stains. *n* = 6. Representative images from six independent experiments. The *p*-*values above the data* denote statistical comparisons of the groups—the control (Ctrl), ischemia–reperfusion injury plus normal saline (IRI+NS), and IRI+FMT groups—which were calculated using ANOVA with Tukey’s *post-hoc* test. ***p < 0.001.

To evaluate the impact of prophylactic FMT, the SDRs were first transplanted with guinea pig feces and then modeled by IRI. It was found that the SDRs in the IRI+FMT group, which were subjected to IRI, had a significantly improved kidney function, as evidenced by the reduced serum urea (**Figure 1A**) and serum Cr levels (**Figure 1B**) compared with the IRI+NS group. To further confirm that prophylactic FMT can reduce kidney damage, histopathological analysis was performed to explore the role of FMT. H&E staining revealed severe tubular epithelial vacuolization, interstitial congestion, and edema in the IRI+NS group, whereas these pathological changes were markedly alleviated in the IRI+FMT group (**Figures 1C, D**), indicating that the model of IRI-induced AKI was successfully established. Evidently, these changes demonstrate that kidney IRI aggravates kidney damage, while prophylactic FMT could protect the kidneys.

### 3.2 FMT reduced local and systemic inflammatory responses

**Figure 2:**
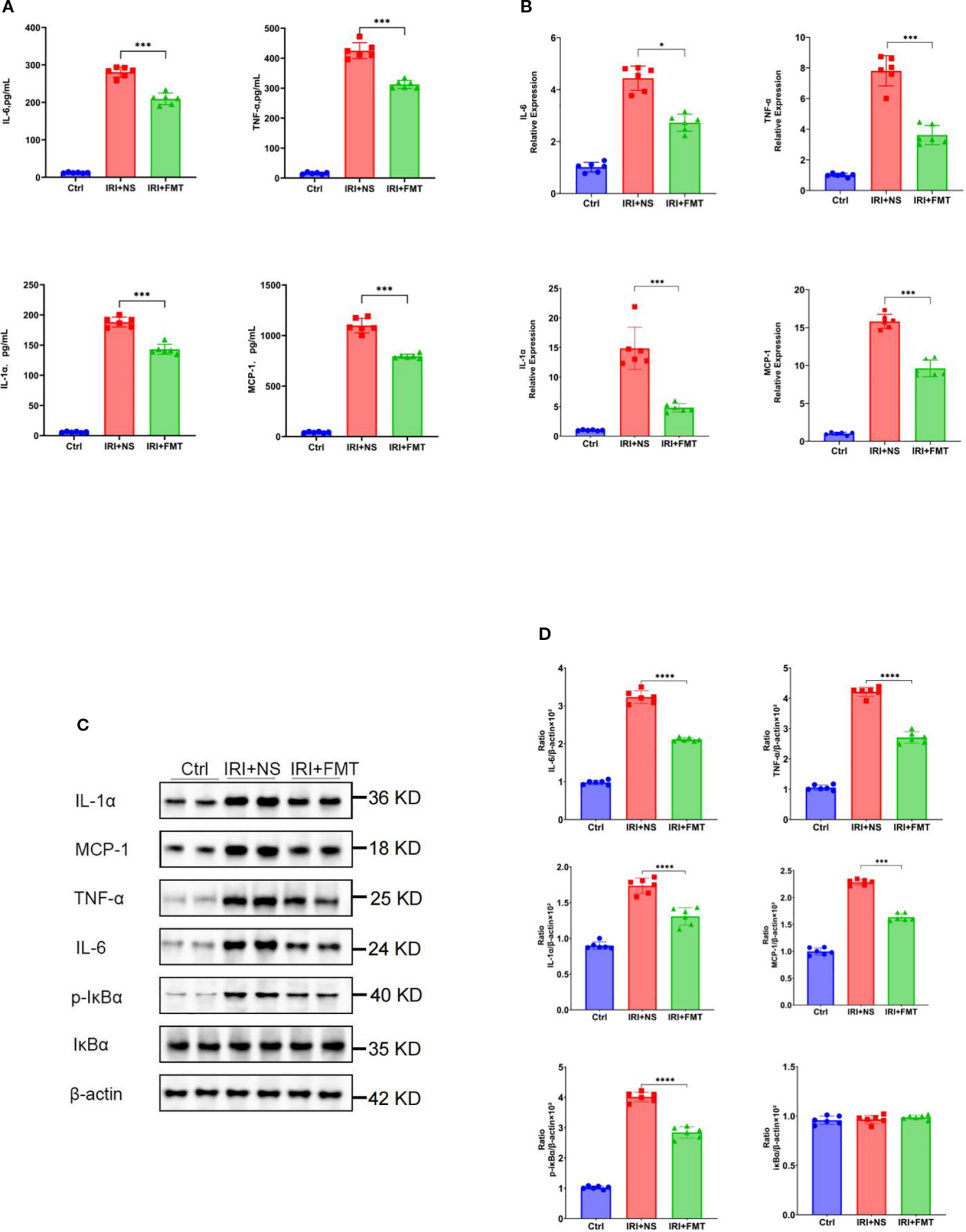
Fecal microbiota transplantation (FMT) reduced systemic or local inflammatory response. **(A)** Protein levels of the pro-inflammatory cytokines and chemokines in the blood. **(B)** mRNA levels of the pro-inflammatory cytokines and chemokines in kidney tissue using real-time PCR. **(C)** Western blot analysis of the pro-inflammatory cytokines in kidney tissue. **(D)** Protein band intensities quantified using optical densitometry and normalized to β-actin levels within the respective experimental groups. *n* = 6. Representative images from six independent experiments. The *p*-values *above the data* denote statistical comparisons on the groups—the control (Ctrl), ischemia–reperfusion injury plus normal saline (IRI+NS), and IRI+FMT groups—which were calculated using ANOVA with Tukey’s *post-hoc* test. *p < 0.05; ***p < 0.001.

Based on the demonstrated efficacy of prophylactic FMT in mitigating kidney injury, we sought to determine its potential to ameliorate inflammatory responses. Prophylactic FMT significantly mitigated both systemic and local inflammatory responses. The serum levels of the pro-inflammatory cytokines (IL-1α, TNF-α, and IL-6) and chemokines (MCP-1) were markedly reduced in the IRI+FMT group compared with the IRI+NS controls (**Figure 2A**), indicating that prophylactic FMT can successfully ameliorate the systemic inflammatory response of SDRs. Due to the reduction of the inflammatory response of the system, we also wanted to know whether prophylactic FMT can reduce the inflammatory response locally. Therefore, real-time PCR was used in the kidney tissue to demonstrate that the IRI+FMT group had decreased mRNA expression of these inflammatory mediators compared with the IRI+NS group (**Figure 2B**). Similar reductions in the expression of the pro-inflammatory cytokines and chemokines in whole kidney lysates from FMT-treated rats were detected using the Western blot assay (**Figures 2C, D**). Furthermore, we were interested in exploring the role of prophylactic FMT in the NF-κB pathway. The Western blot study demonstrated that the IRI+NS group showed an upregulated expression of the phosphorylation of IκBα, whereas the IRI+FMT group showed inhibition of the phosphorylation of IκBα, a key regulator of NF-κB activation, indicating suppression of the inflammatory signaling cascade (**Figures 2C, D**). Altogether, the results indicated that prophylactic FMT might be able to respond through inhibition of the NF-κB pathway to reduce the local and systemic inflammatory responses.

### 3.3 FMT reduced apoptosis and increased tubular proliferating cells

**Figure 3:**
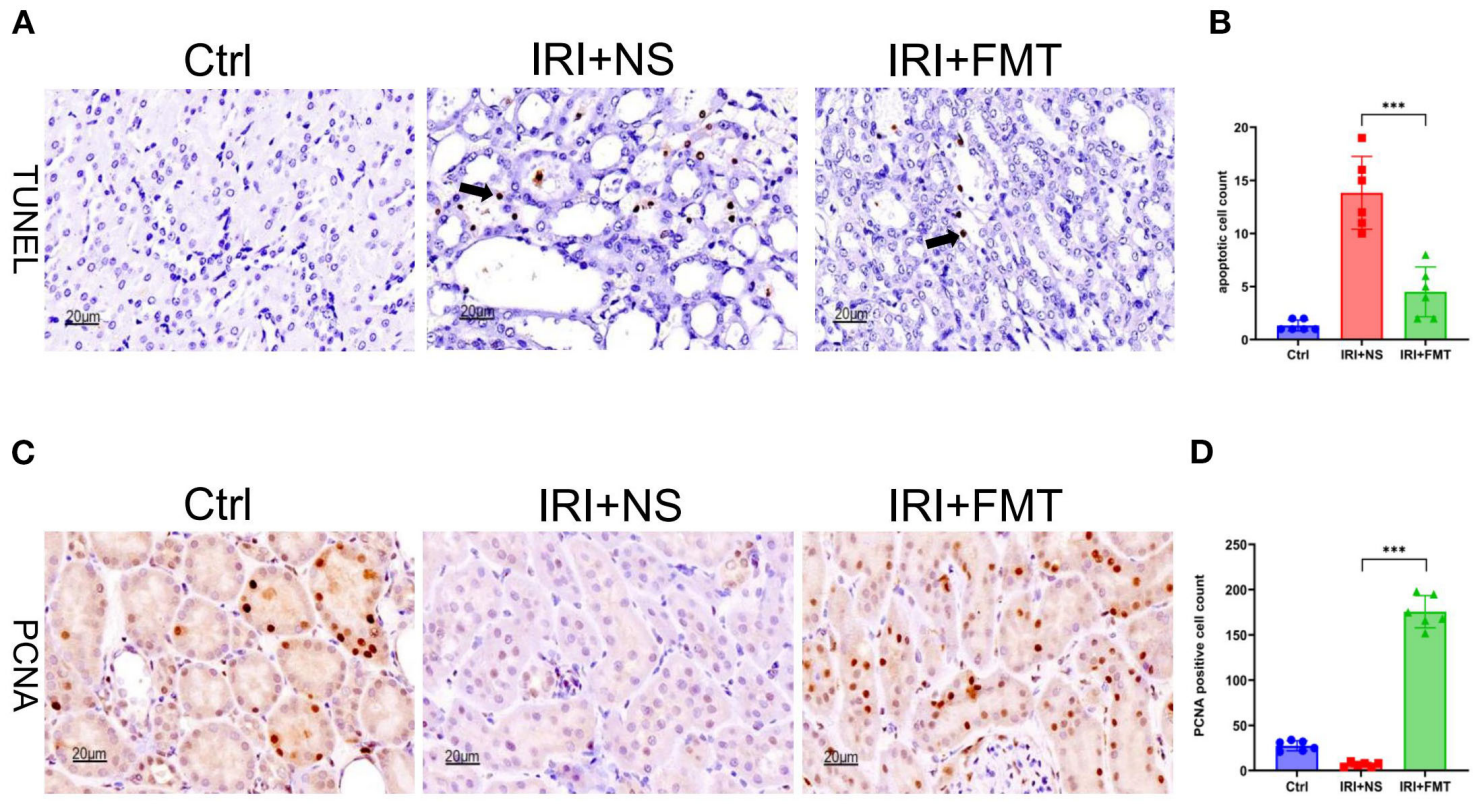
Fecal microbiota transplantation (FMT) reduced apoptosis and increased the proliferation of kidney tubular cells. **(A)** Terminal deoxynucleotidyl transferase dUTP nick-end labeling (TUNEL) staining of apoptotic kidney tubular cells. *Bar*, 20 μm. **(B)** Statistical analysis of TUNEL staining. **(C)** Immunohistochemistry of proliferating cell nuclear antigen (PCNA) in kidney tissue. *Bar*, 20 μm. **(D)** Positive cell counts for PCNA. *n* = 6. Representative images from six independent experiments. The *p*-values *above the data* denote statistical comparisons of the groups—the control (Ctrl), ischemia–reperfusion injury plus normal saline (IRI+NS), and IRI+FMT groups—which were calculated using ANOVA with Tukey’s *post-hoc* test. ***p < 0.001.

Apoptosis is a frequent event in kidney IRI. With the aim of elucidating the potential protective effects of prophylactic FMT on tubular cells apoptosis, we hypothesized that it could alleviate apoptosis. TUNEL staining was used to explore this hypothesis, which revealed significantly fewer apoptotic tubular cells in the IRI+FMT group compared with the IRI+NS group (**Figure 3A**). The statistical graph also demonstrated this phenomenon (**Figure 3B**), indicating that IRI caused apoptosis, whereas prophylactic FMT had the effect of reducing apoptosis. In addition, we investigated whether prophylactic FMT could lead to the proliferation of kidney tubular cells after IRI in the SDR model. Finally, it was observed that kidney tubular cells had proliferated. The PCNA assay showed that the cell proliferation level in the IRI+FMT group was higher than that in the IRI+NS group (**Figure 3C**), and the positive cell count analysis also demonstrated increased proliferation of kidney tubular cells in the IRI+FMT group (**Figure 3D**). In summary, prophylactic FMT could alleviate the impact of apoptosis and increase cell proliferation.

### 3.4 FMT reshaped the gut microbiota and propionic acid positively correlated with Lachnospiraceae

**Figure 4:**
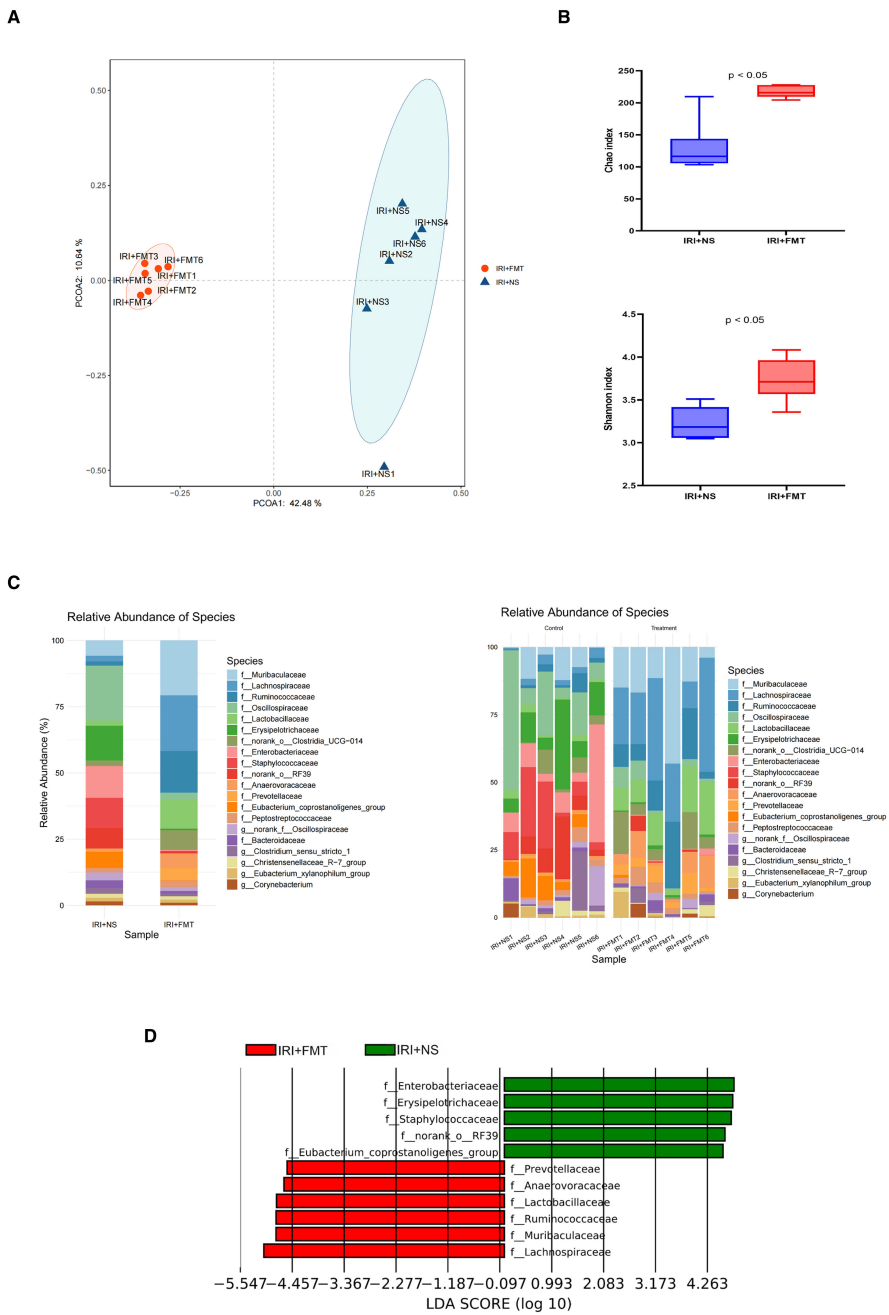
Fecal microbiota transplantation (FMT) altered the microbial community. **(A)** Principal coordinates analysis (PCoA) based on the relative abundance at the family level between groups. **(B)** Shannon index and Chao index in the two groups of rats. The *p*-values indicate comparisons between the ischemia–reperfusion injury plus normal saline (IRI+NS) and IRI+FMT groups, calculated using the Mann–Whitney *U* test (*p* < 0.05). **(C)** Community bar plot analysis showing the composition and the abundance of the different species in all samples. **(D)** Linear discriminant analysis effect size (LEfSe) identifying biologically relevant biomarkers by integrating statistical significance and biological consistency across groups.

To elucidate how prophylactic FMT alleviated IRI, 16S rRNA sequencing was performed, which revealed that FMT altered the gut microbial composition in IRI rats. The results of the beta diversity analyses further separated the fecal microbiota of the IRI+FMT group from the IRI+NS group (**Figure 4A**). Moreover, compared with the IRI+NS group, the samples in the IRI+FMT group were more clustered, indicating that FMT reshaped the gut microbiota of rats. The Shannon diversity index showed a significantly increased evenness (**Figure 4B**) and the same richness results, suggesting that FMT changed the gut microbiome composition in the IRI+FMT group compared with the IRI+NS group. Comparison of the taxonomy profiles demonstrated a marked increase in the relative abundance of Lachnospiraceae at the family level in the IRI+FMT group (**Figure 4C**). Consistently, LEfSe analysis of the fecal microbiota revealed an increase in Lachnospiraceae in the IRI+FMT group compared with the IRI+NS group. Therefore, these findings verified that FMT could reshape the gut microbiota.

**Figure 5:**
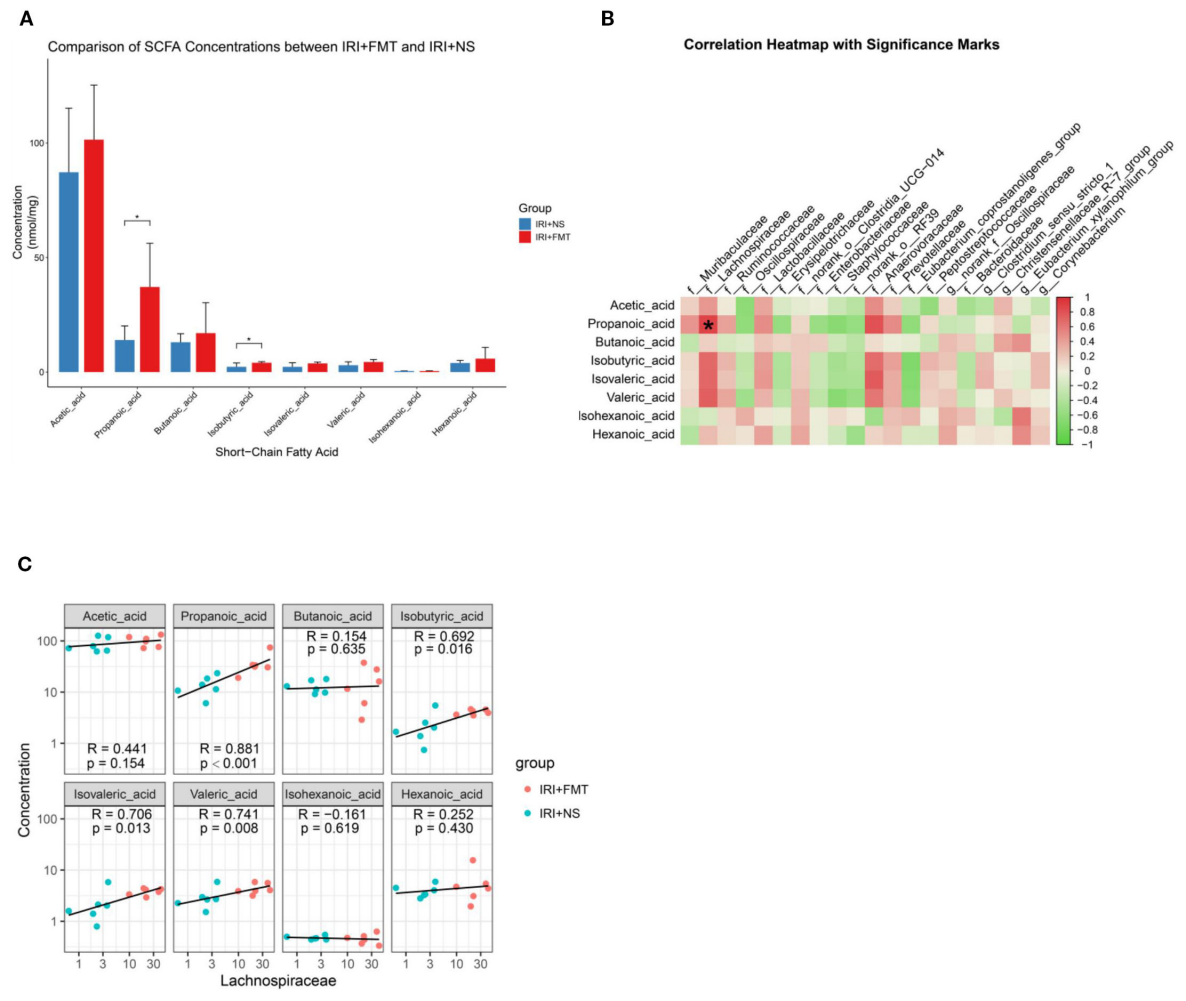
Fecal microbiota transplantation (FMT) increased short-chain fatty acids (SCFAs), and propionic acid was positively correlated with Lachnospiraceae. **(A)** Fecal SCFA quantification in Sprague–Dawley rats (SDRs). The *p*-values indicate comparisons between the ischemia–reperfusion injury plus normal saline (IRI+NS) and IRI+FMT groups, calculated using the Mann–Whitney *U* test. *n* = 6. Representative images from six independent experiments (*p < 0.05). **(B)** Correlation heatmap showing the correlation of the species with the clinical factors (*p < 0.001). **(C)** Scatter plot demonstrating the correlation between propionate levels and Lachnospiraceae abundance.

The gut microbiota can produce SCFAs through intestinal fermentation. Hence, we determined the content of SCFAs in the intestine and found that the levels of propionic acid and isobutyric acid increased significantly after FMT in the IRI+FMT group compared with the IRI+NS group. The levels of other SCFAs increased, but did not show significant differences (**Figure 5A**). Although isobutyric acid had increased, it had a lower content. Therefore, we chose propionic acid in this study. In previous experiments, Lachnospiraceae was found as the most dominant microbiota after FMT. Therefore, we used correlation analysis between the microbiota abundance and the SCFA levels to determine whether Lachnospiraceae is positively correlated with propionic acid (**Figure 5B**). The scatter plot (**Figure 5C**) also proved the same result.

### 3.5 Propionic acid mediated the protective effects of FMT

**Figure 6:**
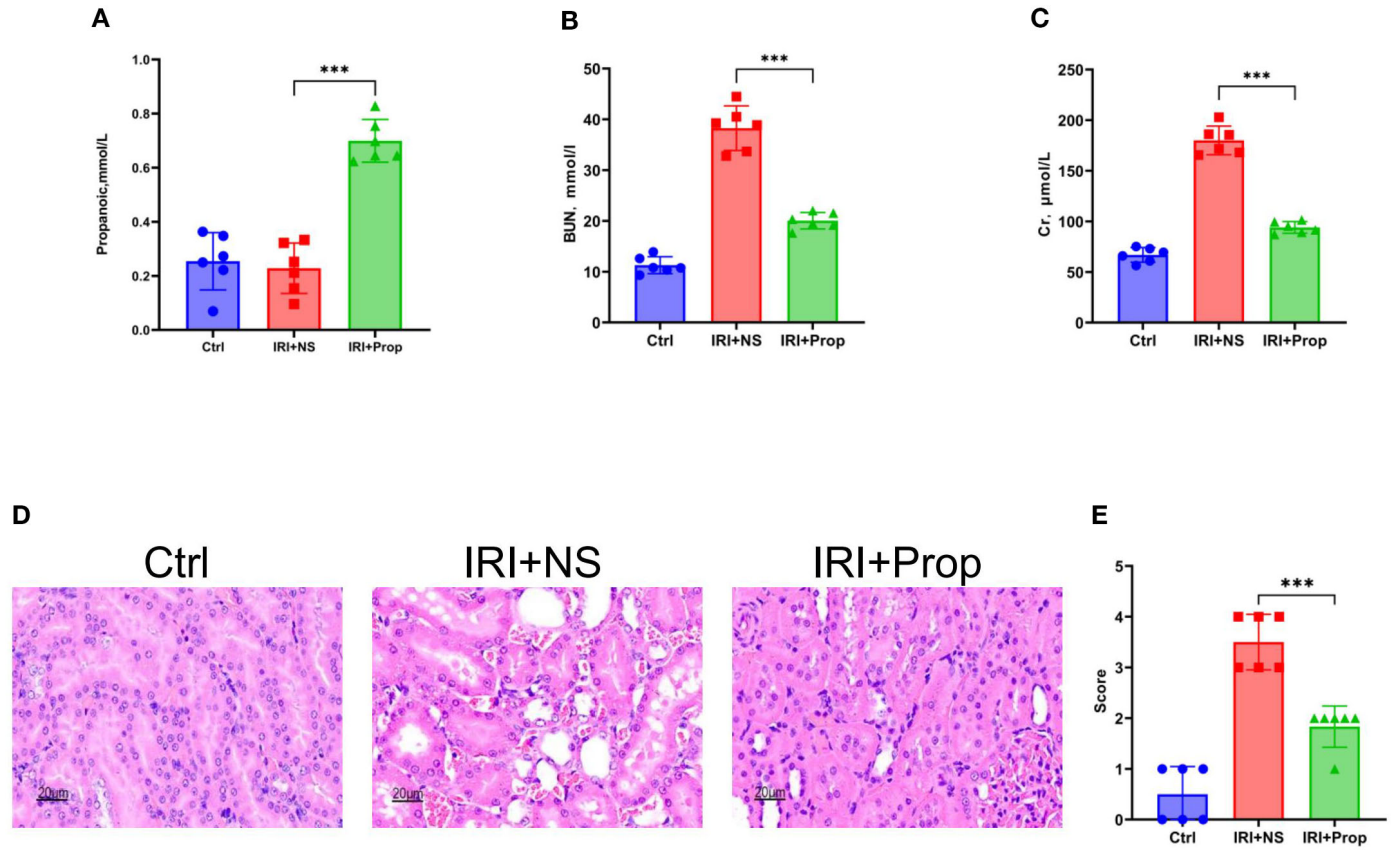
Propionic acid ameliorated kidney damage. **(A)** Propionic acid concentration in rat blood. **(B)** Serum urea nitrogen in rats. **(C)** Serum creatinine in rats. **(D)** Hematoxylin–eosin (H&E) staining of the rats’ kidney sections. *Bar*, 20 μm. **(E)** Pathological scoring of the H&E stains. *n* = 6. Representative images from six independent experiments. The *p*-values *above the data* denote statistical comparisons of the groups—the control (Ctrl), ischemia–reperfusion injury plus normal saline (IRI+NS), and IRI plus propionic acid (IRI+Prop) groups—which were calculated using ANOVA with Tukey’s *post-hoc* test. ***p < 0.001.

To explore the role of propionic acid, we first performed prophylactic feeding of propionic acid to rats and found that the propionic acid levels (**Figure 6A**) in the IRI+Prop group increased compared with that in the IRI+NS group. Prophylactic propionic acid treatment mimicked the renoprotective effects of FMT: the IRI+Prop group exhibited improved serum urea (**Figure 6B**) and serum Cr levels (**Figure 6C**) and diminished histopathological damage (**Figures 6D, E**), indicating that the prophylactic propionic acid treatment protected kidney function.

**Figure 7:**
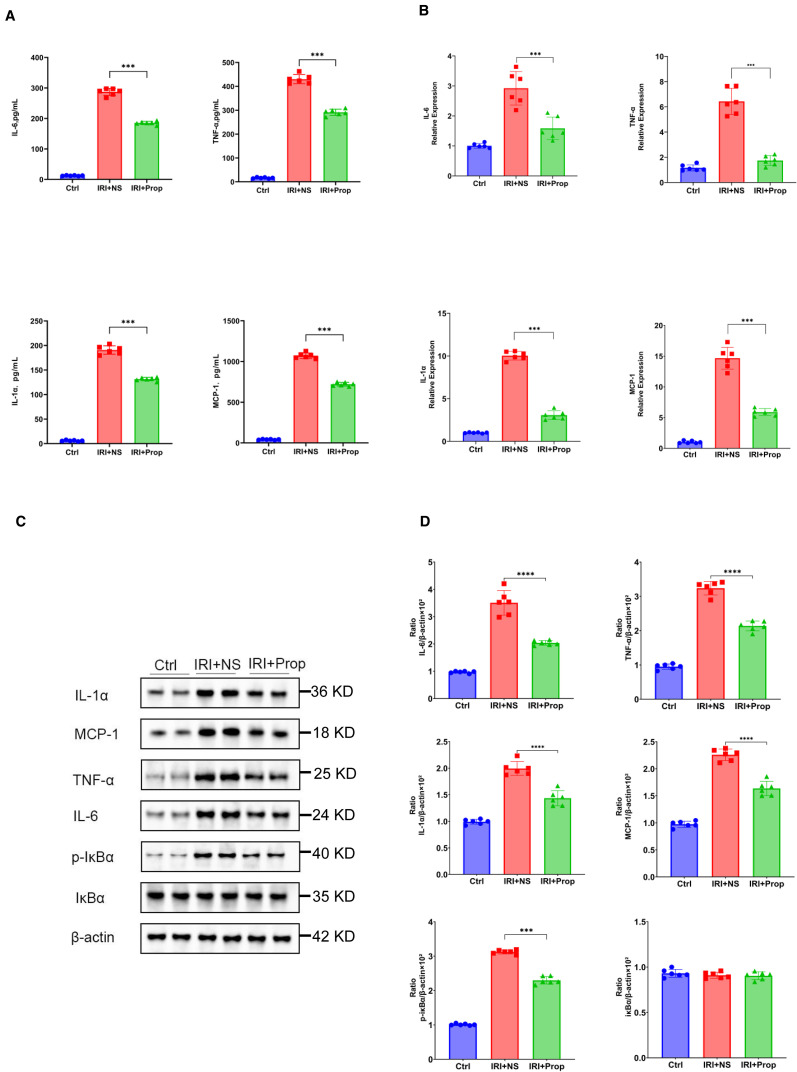
Propionic acid reduced the systemic or local inflammatory response. **(A)** Protein levels of the pro-inflammatory cytokines and chemokines in the blood. **(B)** mRNA levels of the pro-inflammatory cytokines and chemokines in kidney tissue using real-time PCR. **(C)** Western blot analysis of the pro-inflammatory cytokines in kidney tissue. **(D)** Protein band intensities quantified using optical densitometry and normalized to β-actin levels within the respective experimental groups. *n* = 6. Representative images from six independent experiments. The *p*-values *above the data* denote statistical comparisons of the groups—the control (Ctrl), ischemia–reperfusion injury plus normal saline (IRI+NS), and IRI plus propionic acid (IRI+Prop) groups—which were calculated using ANOVA with Tukey’s *post-hoc* test. **p < 0.01; ***p < 0.001.

Through our research, we found that, after prophylactic propionic acid treatment in the rat IRI model, the serum levels of the pro-inflammatory cytokines (IL-1α, TNF-α, and IL-6) and chemokines (MCP-1) reduced the systemic inflammation response (**Figure 7A**) in the IRI+Prop group compared with the IRI+NS group. For local inflammatory response, the same result was assayed using Western blot and real-time PCR (**Figures 7B–D**). In addition, the expression of the phosphorylation of IκBα also decreased in the IRI+Prop group compared with the IRI+NS group (**Figures 7C, D**), as it is the same as prophylactic FMT. In conclusion, prophylactic propionic acid treatment reduced the local and systemic inflammatory responses by inhibiting the NF-κB pathway.

**Figure 8:**
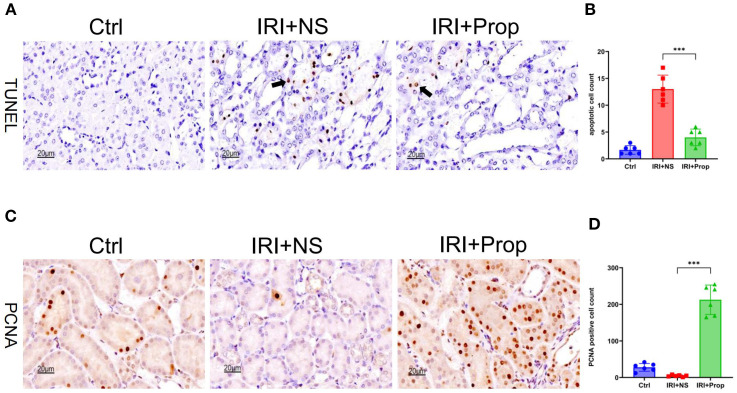
Propionic acid reduced apoptosis and increased the proliferation of kidney tubular cells. **(A)** Terminal deoxynucleotidyl transferase dUTP nick-end labeling (TUNEL) staining of apoptotic tubular cells. *Bar*, 20 μm. **(B)** Statistical analysis of TUNEL staining. **(C)** Immunohistochemistry of proliferating cell nuclear antigen (PCNA) in kidney tissue. *Bar*, 20 μm. **(D)** Positive cell counts for PCNA. *n* = 6. Representative images from six independent experiments. The *p*-values *above the data* denote statistical comparisons of the groups—the control (Ctrl), ischemia–reperfusion injury plus normal saline (IRI+NS), and IRI plus propionic acid (IRI+Prop) groups—which were calculated using ANOVA with Tukey’s *post-hoc* test. ***p < 0.001.

Similarly to prophylactic FMT, TUNEL staining was used. It was found that the apoptosis of tubular cells was reduced in the IRI+Prop group compared with the IRI+NS group (**Figures 8A, B**). The PNCA assay demonstrated an enhanced tubular cell proliferation in the IRI+Prop group compared with the IRI+NS group (**Figures 8C, D**).

In summary, the main reason for the effect of prophylactic FMT on kidney IRI is the role of propionic acid, a product of gut microbes.

### 3.6 Propionic acid exerted effects via GPR43 signaling

**Figure 9:**
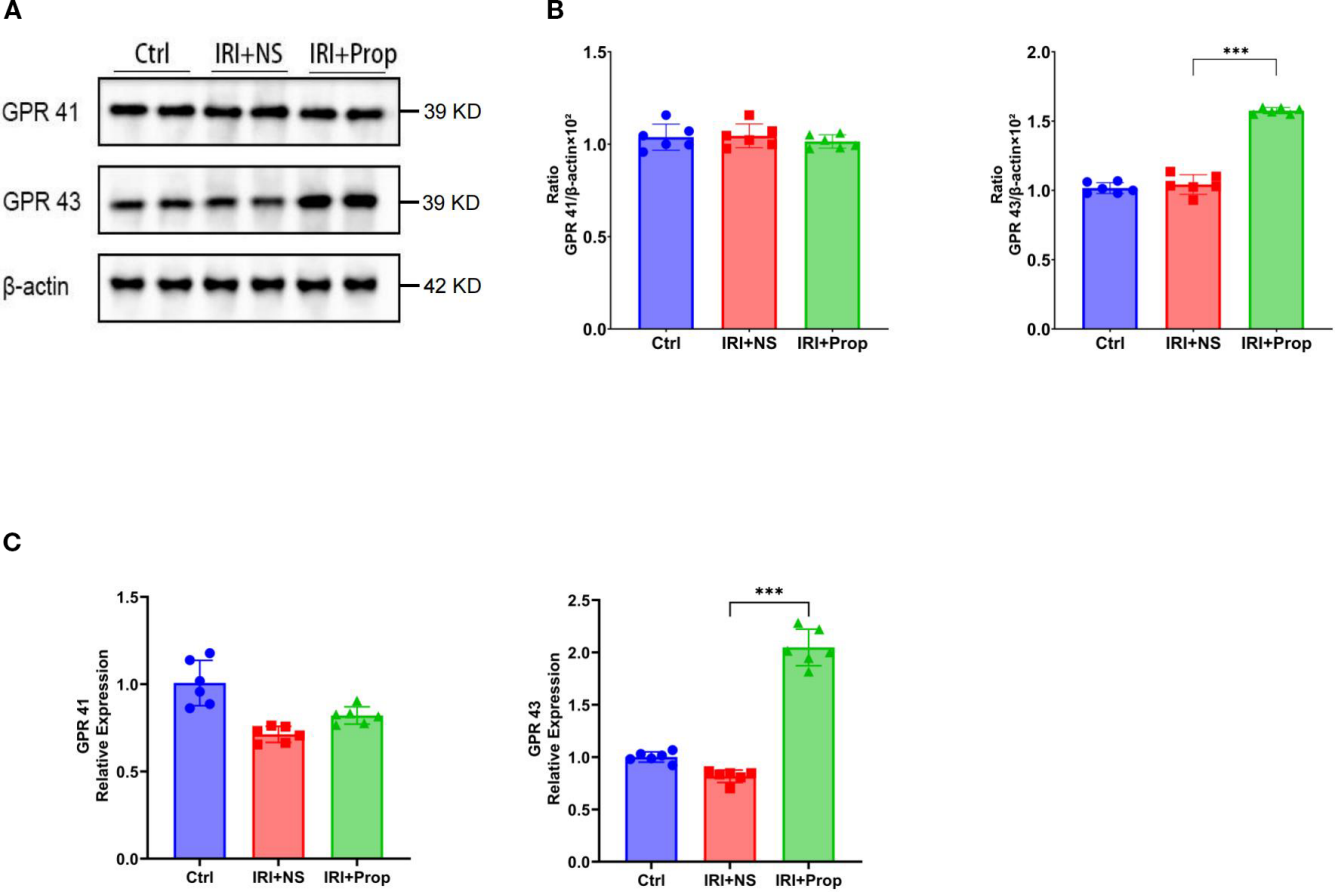
Propionic acid upregulated GPR43 expression. **(A)** Western blot analysis of GPR41 and GPR43. **(B)** GPR41/β-actin and GPR43/β-actin ratios for each band per group determined using the optical density method and subsequently normalized to β-actin levels. **(C)** Real-time PCR of GPR43 and GPR41 in the kidney tissues of rats with kidney injury treated with propionic acid. *n* = 6. Representative images from six independent experiments. The *p*-values *above the data* denote statistical comparisons of the groups—the control (Ctrl), ischemia–reperfusion injury plus normal saline (IRI+NS), and IRI plus propionic acid (IRI+Prop) groups—which were calculated using ANOVA with Tukey’s *post-hoc* test. ***p < 0.001.

GPRs are a mechanism of action for SCFAs. The Western blot analysis revealed an enhanced expression of GPR43 in the IRI+Prop group compared with the IRI+NS group, but no difference in the expression of GPR41 (**Figures 9A, B**), after propionic acid. Furthermore, real-time PCR confirmed a concomitant upregulation of the mRNA levels of GPR43 (**Figure 9C**). This suggests that propionic acid may act through GPR43 rather than GPR41.

## 4 Discussion

The pathophysiology of kidney IRI encompasses a multifactorial cascade dominated by dysregulated inflammatory pathways that critically drive AKI pathogenesis. The clinical trajectory of AKI is particularly dire, exhibiting elevated risks of both acute mortality and long-term sequelae, including an accelerated progression to CKD and end-stage kidney failure (Rewa and Bagshaw, 2014; Liu and Zhang, 2021; Li et al., 2023; Xu et al., 2024). Consequently, therapeutic interventions targeting IRI-mediated AKI represent an urgent unmet need. This study delineated three results underpinning FMT-mediated renoprotection in IRI. Firstly, the intra-group comparisons revealed that IRI+FMT had significantly lowered serum Cr and urea nitrogen levels relative to the IRI+NS controls, paralleled by histopathological attenuation of tubular damage. Secondly, a reduction of the inflammatory response occurred in the IRI+FMT group, and the phosphorylation of IκBα was reduced. Thirdly, the kidney tubular cell apoptosis was significantly reduced in the IRI+FMT group. Enhanced proliferative activity, as evidenced by the increase in PCNA+ cells, further underscored the pro-regenerative potential of FMT. The therapeutic efficacy of FMT primarily stemmed from gut microbiota remodeling in SDRs, which increased the circulating level of the microbial metabolite propionic acid—a mechanistic mediator of renoprotection in IRI. This conclusion was reinforced by parallel experiments demonstrating that prophylactic propionic acid administration recapitulated the protective effects of FMT. In this study, real-time PCR and Western blot analyses further demonstrated that the GPR41 expression exhibited comparable levels in both the IRI+NS and IRI+Prop groups, while the GPR43 expression was significantly upregulated in response to propionic acid. Therefore, we propose that propionic acid plays a significant role in the effects of FMT.

Propionic acid, the most common SCFA, could be used as a potential treatment for the disease. Previous studies have demonstrated that propionic acid can alleviate collagen-induced arthritis in mice (Bai et al., 2021). Propionic acid administration significantly attenuated cardiac hypertrophy, fibrosis, vascular dysfunction, and hypertension in two different mouse models of hypertensive cardiovascular injury (Bartolomaeus et al., 2019). Notably, in a study on CKD, the SCFA levels in patients with CKD were measured at different stages, which found that the propionic acid levels in these stages had varying degrees of decline. Finally, in a preclinical mouse model of transition from AKI to CKD induced by the use of folic acid, treatment with propionic acid attenuated kidney injury, suppressed the expression of pro-inflammatory, and facilitated long-term recovery of renal function (Corte-Iglesias et al., 2024). In this study, propionic acid, which was the focus of the study, also significantly provided a protective effect on the kidneys.

Inflammation represents hallmark pathological features of this experimental model, with propionic acid intervention demonstrating inhibitory effects on this process. Inflammation manifests as a sophisticated biological response orchestrated by local and systemic reactions to diverse immunological and non-immunological stimuli. The NF-κB signaling cascade, a critical mediator of inflammatory responses, as evidenced in numerous studies (Liu et al., 2017), demonstrates reduced activity in the majority of investigations involving SCFAs. Notably, experimental evidence reveals that butyrate inhibits NF-κB signaling through preventing the proteasome-mediated breakdown of ubiquitinated IκB inhibitors (Martin-Rodriguez et al., 2015). A recent study suggested that, similar to butyrate, propionate may inhibit the activation of NF-κB in colonocytes, thereby reducing the expression of pro-inflammatory factors in colon tissue (Li et al., 2024a). Recent advances have established that SCFA can bind to GPRs (e.g., GPR41 and GPR43) (van der Hee and Wells, 2021). Another study also reported that SCFAs, in particular propionic acid, are important signaling molecules that act as GPR activators and histone deacetylase (HDAC) inhibitors (Zheng et al., 2019). In addition, GPR43 on colonic T cells potentially induces the differentiation of regulatory T cells (Tregs) when binding to SCFAs. Importantly, propionate or SCFA mix-treated Rag^−/−^ mice injected with naive T cells and Tregs had lower level of colitis than mice that received water (Brown et al., 2003). This suggests that propionic acid can act as an agonist of GPR43. Therefore, propionic acid inhibits NF-κB signaling and reduces inflammation, possibly through GPR43.

In kidney IRI, apoptotic mechanisms profoundly influence pathology, with tubular epithelial cells demonstrating a marked vulnerability to apoptotic stimuli (Liu et al., 2022). Both fully damaged and sub-lethally injured cells, accompanied by shed apical membrane components and cytoplasmic debris, accumulate in the tubular lumen. These aggregates coalesce into obstructive casts, directly contributing to tubular occlusion and functional impairment (Havasi and Dong, 2016). To date, in the kidney IRI model, there have been several research studies for its treatment. Targeted therapeutic modalities can ameliorate IRI-triggered apoptotic cascades in renal tubular cells. For example, glutamine leads to the transcriptomic and proteomic reprogramming of kidney tubules in mice, leading to a reduction in apoptosis (Thomas et al., 2022). Empagliflozin ameliorated kidney IRI in a mouse IRI model by promoting the AMPK-OPA1 pathway to reduce inflammation and enhance mitochondrial fusion, and in the process was shown to decrease tubular apoptosis (Yang et al., 2023). On the other hand, decreased levels of apoptosis and increased levels of the ATG-7 protein with SCFA treatment indicated that SCFAs can induce cellular autophagy and thus delay apoptosis (Andrade-Oliveira et al., 2015). In this study, it was demonstrated that kidney IRI apoptosis could be reduced by prophylactic propionic acid treatment. This represents the potential of SCFA treatment in reducing kidney tubular apoptosis.

Kidney IRI could be divided into an initiation phase and an extension phase, followed by a recovery phase. The recovery phase is characterized by an increase in dedifferentiated and mitotic cells in the damaged renal tubules (Zahedi et al., 2004). It has been previously demonstrated that kidney repair is associated with a cascading response of dedifferentiation, proliferation, and the migration of surviving epithelial cells, which may also include mesenchymal stem cells, to replace dead cells after injury (Guo and Cantley, 2010; Bonventre and Yang, 2011; El Sabbahy and Vaidya, 2011). It has been shown that SCFAs induced hepatocyte-like organ growth in a mouse hepatic resection model and mouse liver SCD1 expression, which in turn led to hepatocyte proliferation (Yin et al., 2023). Moreover, SCFAs inhibited glomerular cell apoptosis and promoted proliferation in a model of chronic kidney failure and a model of adriamycin-induced focal segmental glomerulosclerosis (Zha et al., 2023). In addition, our study found an increase in PNCA+ cells after prophylactic propionic acid treatment, and PCNA is a recognized biomarker of DNA synthesis. These observations collectively suggest that SCFAs might accelerate the transition of damaged renal tubules into the recovery phase, thereby driving the expansion of PCNA-positive cell populations through enhanced cellular proliferation mechanisms.

Alteration of the diversity of the gut microflora in SDRs after FMT is an expected result, as demonstrated in our study, which revealed an increase in the relative abundance of Lachnospiraceae and a positive correlation with the expression of propionic acid production. Although the increase in the abundance of Muribaculaceae was significant, the correlation with SCFAs was less significant. Lachnospiraceae produces propionic acid through two pathways: the propylene glycol pathway and the acrylate pathway (Vacca et al., 2020). Genomic analysis of the acrylate pathway revealed that lactyl-CoA dehydratase sequences serve as the most suitable marker genes for this metabolic route. Notably, members of Lachnospiraceae also harbor lactyl-CoA dehydratase genes, enabling the production of both butyrate and propionate through distinct metabolic branches. The *pduP* gene encodes a CoA-dependent propionaldehyde dehydrogenase that catalyzes the conversion of propionaldehyde to propionyl-CoA. This gene has been investigated for its suitability as a biomarker of the propanediol pathway, which utilizes carbohydrates such as fucose and rhamnose for propionate production (Seeliger et al., 2002; Scott et al., 2006). By assessing the bacterial growth and fermentation product formation in the presence of these sugars compared to glucose, another study analyzed the metagenomic database and found that the *pduP* gene is widespread in Lachnospiraceae (Qin et al., 2010; Reichardt et al., 2014), confirming the propanediol pathway as functionally active and significantly enriched within members of Lachnospiraceae. Therefore, it is possible that the production of propionic acid in Lachnospiraceae is through the propylene glycol pathway. Lachnospiraceae comprise specialized anaerobic microorganisms abundant in the human gut. They produce SCFAs and secondary bile acids, which are essential for the maintenance of gut integrity, microbial homeostasis, immune function, and energy balance. As a result, Lachnospiraceae species are generally considered to be beneficial microorganisms (Sorbara et al., 2020). While the physiological regulatory effects and pathological mechanisms exerted by the gut microbiota on the host are predominantly mediated through their metabolites rather than direct bacterial interactions, prophylactic FMT has emerged as a promising therapeutic strategy for the prevention of kidney IRI.

In FMT studies, oral gavage administration could cause the intestinal flora to be affected by stomach acid. To address this limitation, our investigative protocol employed an enteric-coated capsule formulation designed to mitigate the effect of the acidic environment of the upper gastrointestinal tract. This approach has demonstrated significant advantages in preserving microbial activity, as the protective capsule ensures enhanced survival of probiotic bacteria during gastric transit while maintaining intestinal colonization efficacy. This protocol is also essential for the issue of security, in which patients are given fecal suspension via a nasogastric tube (or nasoduodenal tube) or colonoscopy (Kim and Gluck, 2019). This FMT method is associated with safety risks and is relatively invasive, which can be a barrier to patient acceptance. Thus, oral capsules could eliminate the disadvantages of this aspect.

The prophylactic success of FMT in this study underscored its potential for clinical translation, particularly in high-risk settings such as kidney transplantation, cardiovascular disease, post-CPR syndrome, or sepsis. Our earlier work established the utility of FMT in nephrolithiasis (Wang et al., 2023), and this study extended its applicability to ischemic AKI—a condition lacking targeted therapies. By restoring the gut microbial diversity and enriching beneficial taxa such as Lachnospiraceae, FMT might counteract the impacts commonly observed in AKI. Propionic acid supplementation offered a more scalable alternative, circumventing the complexities of live microbiota transfer. Its efficacy via oral administration further supports its viability as a nutraceutical or adjunctive therapy. However, clinical translation of propionic acid-based therapies is impeded by two key challenges: its short half-life, which necessitates frequent dosing and limits sustained bioactivity, and its gastrointestinal side effects—including mucosal irritation, altered motility, and dysbiosis—arising from rapid local accumulation. To address these, slow-release formulations have emerged as a viable strategy: propionic acid encapsulation in pH-sensitive polymers (e.g., Eudragit^®^) or lipid-based nanoparticles mitigate gastric irritation while prolonging systemic exposure (Zain et al., 2022). Prebiotic approaches (e.g., high-fiber diets) to enrich endogenous propionic acid-producing taxa also warrant exploration. This design not only extends the therapeutic duration and minimizes the dosing frequency but also mitigates acute irritation, enhancing patient tolerability. In addition, pH-sensitive or enzyme-responsive carriers could further refine release, targeting propionic acid delivery to specific intestinal regions (e.g., distal colon) where its anti-inflammatory or metabolic benefits are maximal, thereby bridging preclinical potential to safer, clinically applicable interventions.

While this study advances our understanding of the gut microbiota in IRI, key limitations must be addressed to clarify translational potential. ​Firstly, the therapeutic utility of FMT in IRI remains uncharacterized, as the current work focused on preventive administration (pre-IRI), neglecting clinical scenarios requiring (post-IRI) intervention. Studies evaluating delayed FMT efficacy—mitigating IRI complications post-onset—are critical. ​Secondly, long-term IRI outcomes (e.g., progressive fibrosis, aberrant remodeling, and sustained functional impairment) are unexamined, and existing data focused on acute inflammation and early damage, leaving gaps in the durability of the benefits and delayed risks. Thirdly, existing preclinical models, predominantly rats and guinea pigs, may not sufficiently recapitulate the complexity of the human microbiome or this specific host–microbe interaction—particularly due to evolutionary divergence in conserved microbial pathways such as propionic acid synthesis, evidenced by the differential prevalence of the *pduP* gene in human-associated Lachnospiraceae *versus* its absence/divergence in guinea pig microbiota (Sorbara et al., 2020)—limiting clinical inference. Therefore, humanized microbiota models will be essential to validate translational potential. Finally, while conserved metabolic pathways support Lachnospiraceae-derived propionic acid production (Reichardt et al., 2014), future studies using mono-colonized gnotobiotic models or bacterial isolates are needed to confirm direct causality. Similarly, the necessity of GPR43 must be verified via receptor blockade or genetic ablation.

In conclusion, prophylactic FMT modulated the gut microbiota composition, resulting in the increase of propionic acid, a critical gut-derived microbial metabolite. This intervention demonstrated significant renoprotective effects through the attenuation of tubular injury and the preservation of the kidney functional integrity, suggesting its therapeutic potential in the prevention of kidney IRI.

## Data Availability

The datasets of this study were deposited in the National Center for Biotechnology Information under Bio Project accession code PRJNA1215045.
